# Supplementary material for: Structure of the recombinant RNA polymerase from African Swine Fever Virus
Source: Nat Commun. 2024 Feb 21;15:1606. doi: 10.1038/s41467-024-45842-7 (PMC10881513; doi:10.1038/s41467-024-45842-7)
Supplement: Supplementary file 1 — Supplementary Information [file 41467_2024_45842_MOESM1_ESM.pdf]

Supplementary Information

Supplementary Figures

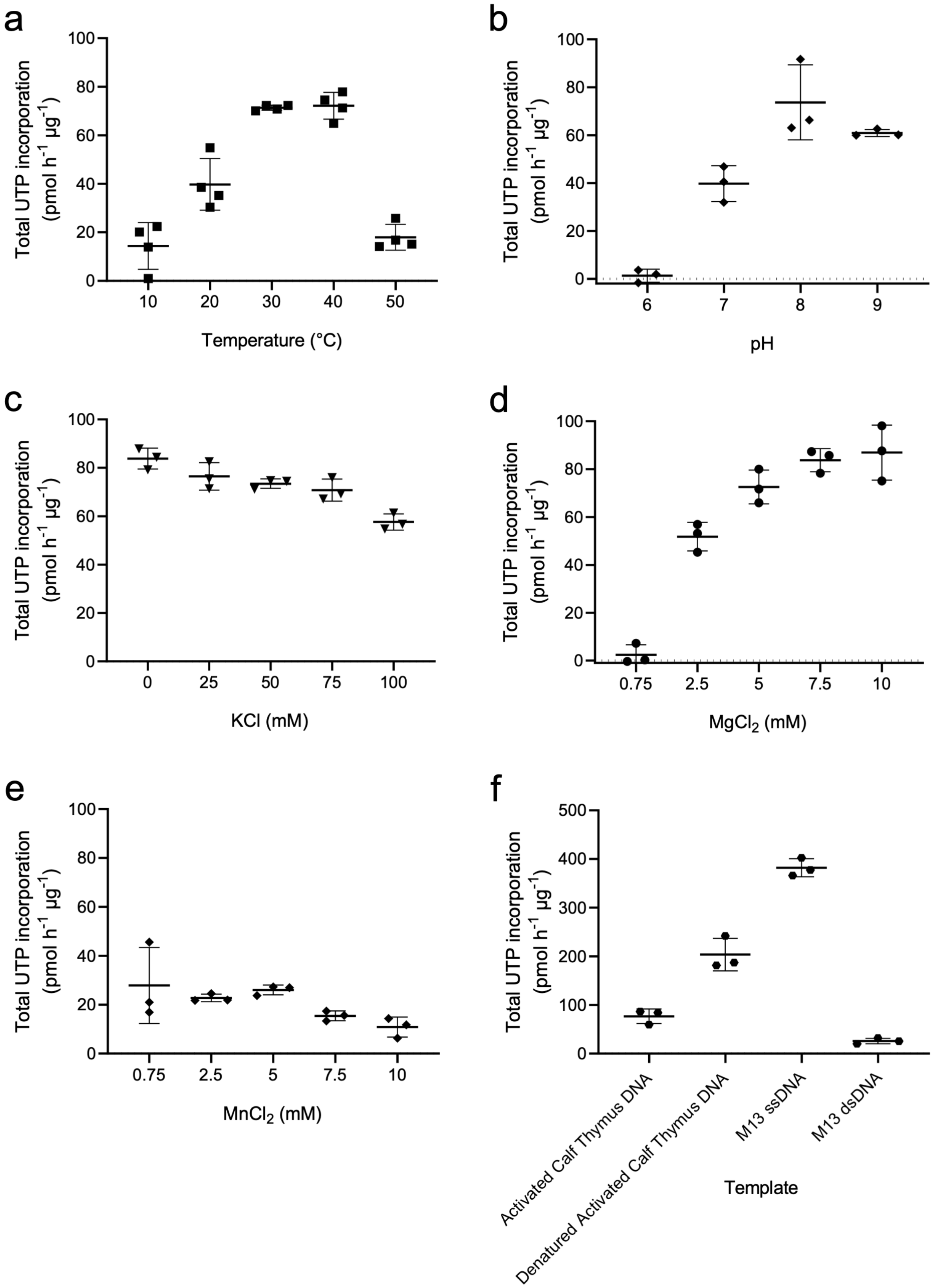

Supplementary Figure 1. Optimization of nonspecific *in vitro* transcription assay. Reactions with recombinant ASFV RNAP measured in triplicates over a range of **a)** temperatures (10-50°C), **b)** pH (6-9), **c)** addition of KCl (0 - 100 mM), and divalent cation, **d)** using $MgCl_2$ (0.75-10 mM) or **e)** $MnCl_2$ (0.75-10 mM) as indicated in each graph. **f)** Different DNA transcription templates were compared using 250 ng of either activated calf thymus DNA in native or heat denatured form, and M13mp18 circular DNA in native single stranded or RF I dsDNA form. The activity was calculated measuring the incorporation of radiolabeled [α-^{32}P]-UTP and normalized

against the negative controls without RNA polymerase and reported as the mean for each triplicate with corresponding standard deviation. Source data are provided as a Source Data file. All experiments were independently repeated three times in similar buffer conditions providing similar results. The complete list of buffer conditions used for the assays is reported in Methods.

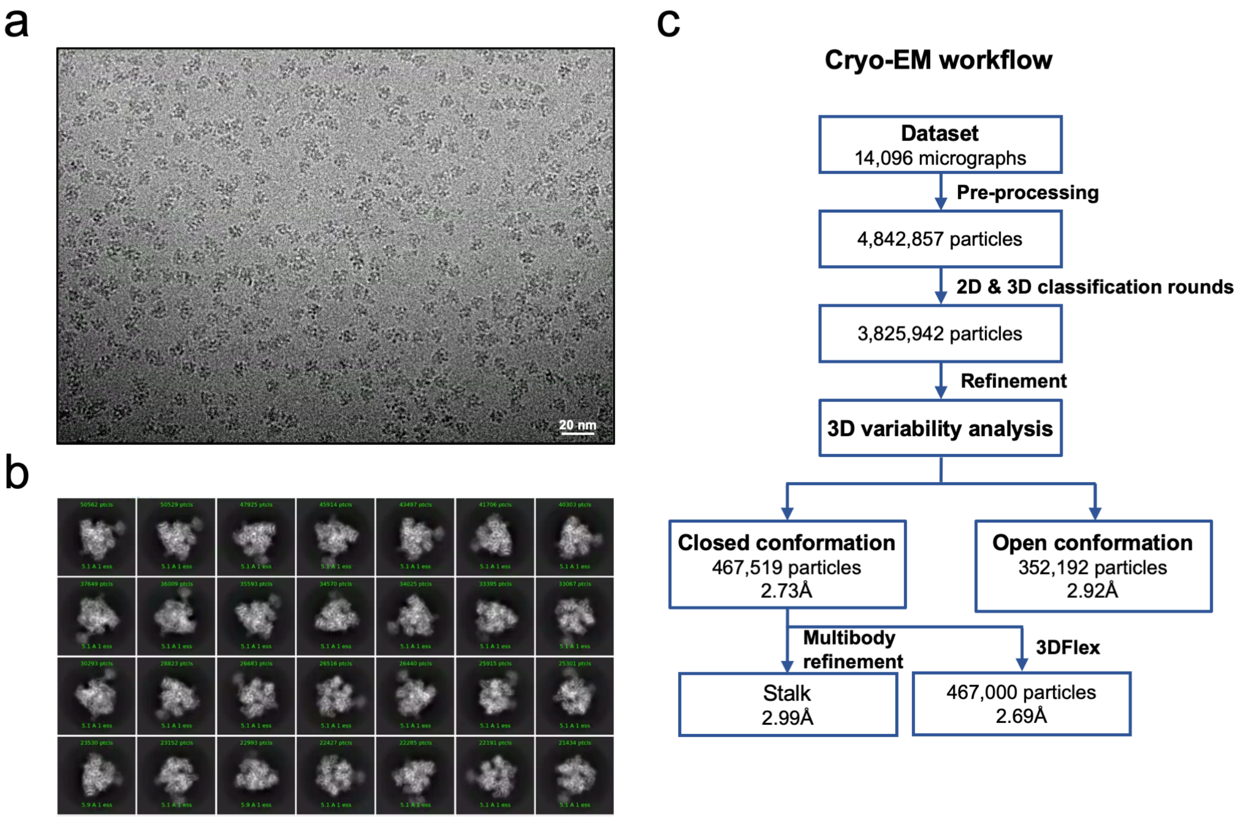

Supplementary Figure 2. Cryo-EM data results and processing workflow. a) Representative motion-corrected cryo-EM micrograph. **b)** Selected 2D projections of the RNA polymerase obtained from cryoSPARC v3.0 [1], showing on the top the number of particles contained in each class, the resolution achieved (in Å), and a class quality evaluation on the bottom with the lowest value 1 having the highest homogeneity. **c)** Schematic of the data processing pipeline reporting the main results obtained.

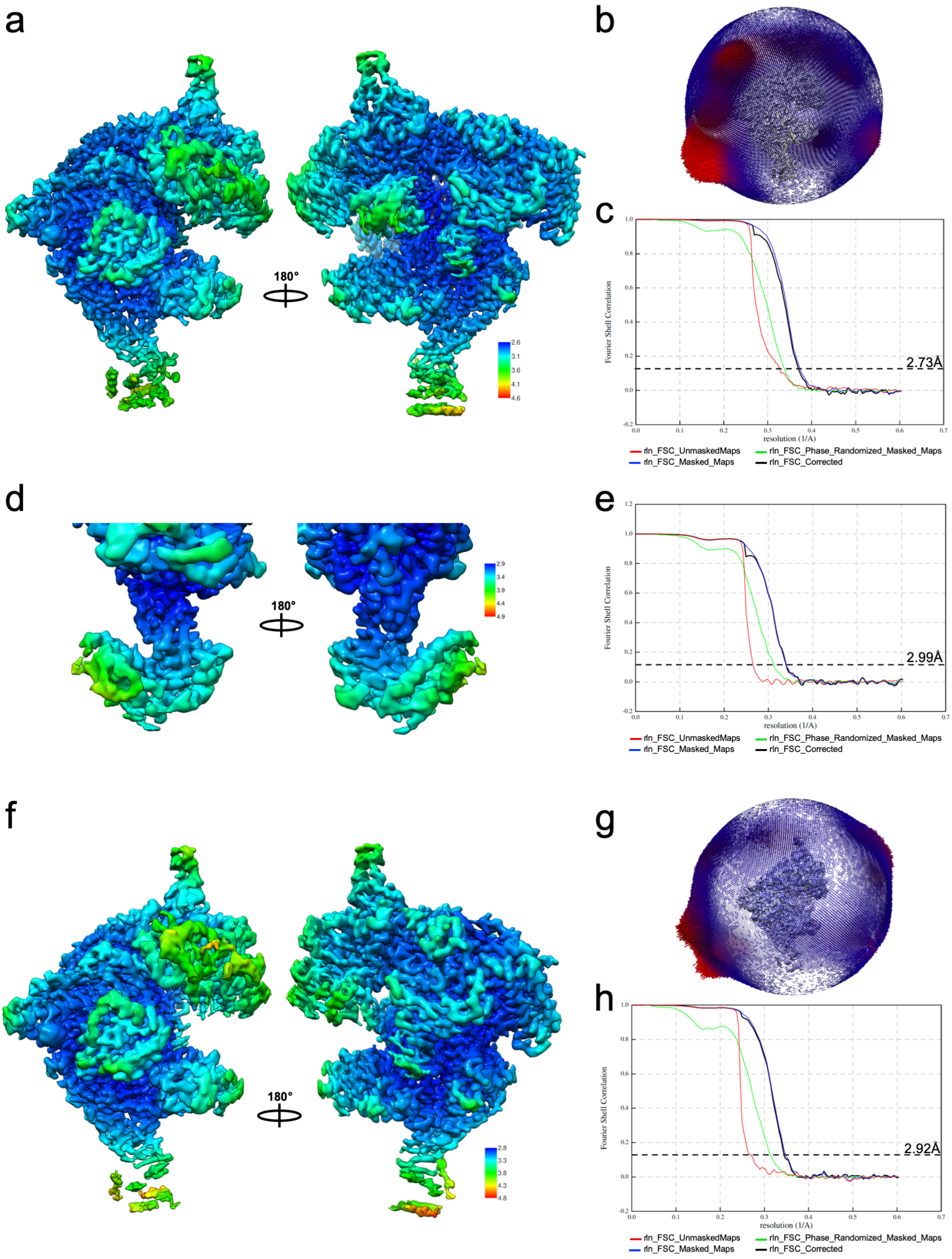

Supplementary Figure 3. Map quality evaluation of the ASFV core RNAP in the closed and open conformations. a, d, f) Local resolution variation of the 3D reconstructions after map sharpening; the maps are presented in two orientations and coloured based on local resolution as indicated in the corresponding scale bar. The maps correspond to ASFV RNAP in closed (**a**) and open (**f**) conformations, and to the stalk domain (**d**) after multibody refinement (Relion v4 [2]). **b, g)** Angular distribution plots illustrating the contribution of the number of particles to the final 3D reconstruction. The plot is shown as a sphere around the EM map (in grey), the particles are shown as blue bars in all orientations where they are homogenously represented, while the bars are depicted in red in all over-represented angles. The plots in (**b**) and (**g**) corresponds to closed and open conformations, respectively. **c, e, h**) Gold Standard Fourier Shell Correlation (FSC) plot obtained from post processing in Relion v4. The dashed line represents a 0.143 cut-off used to define the overall nominal resolution of the map which corresponds to the corrected curve (in black). For the curve labelled as masked, the FSC was calculated generating a mask with soft edges.

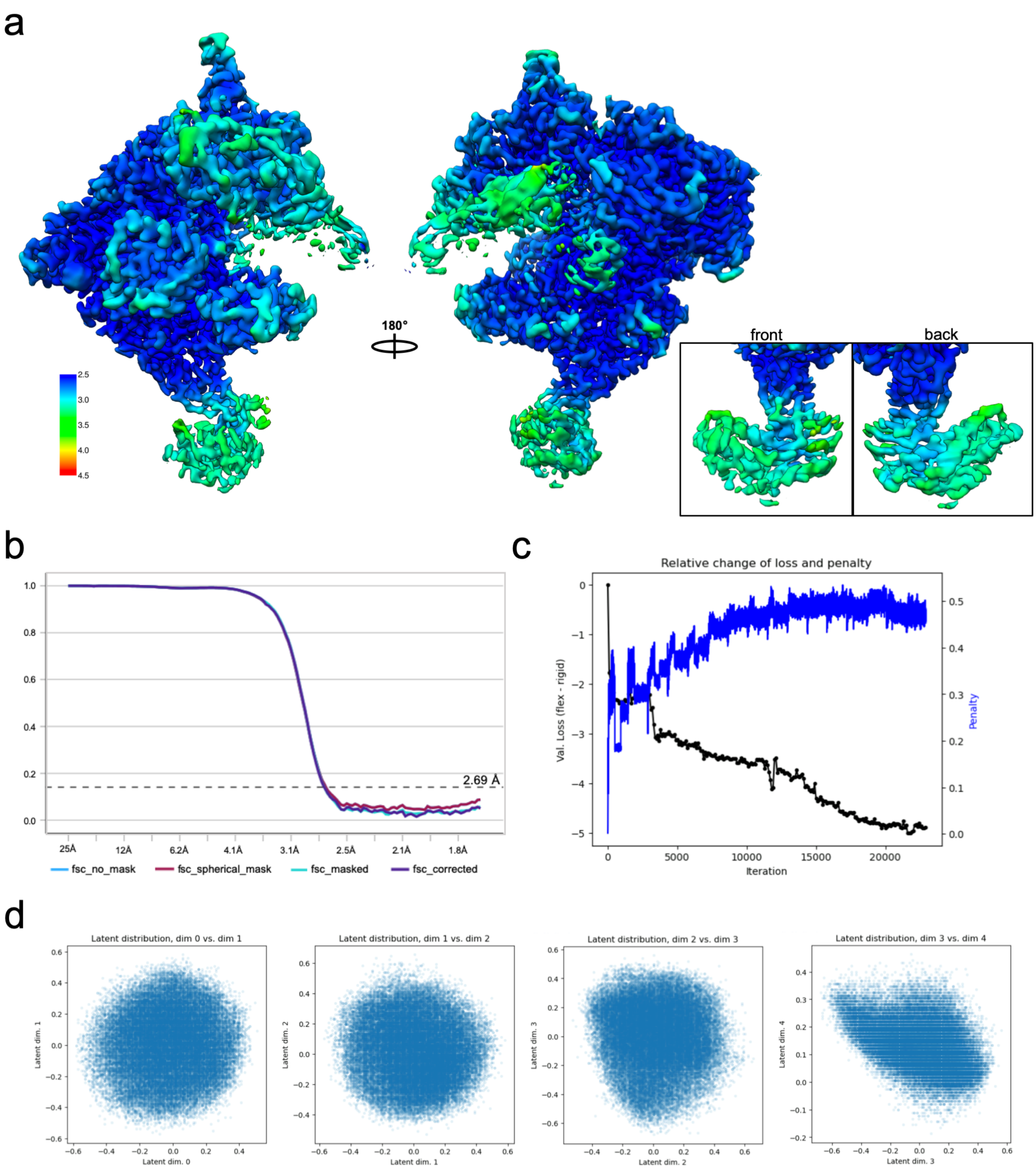

Supplementary Figure 4. Map quality evaluation and validation plots for the 3DFlex refinement. a) Local resolution variation of the 3D reconstruction. The map is presented in two orientations and coloured based on local resolution as indicated in the corresponding scale bar. Enlargements of the stalk are shown to allow a better comparison with the multibody refinement result in Supplementary Figure 3d. **b)** FSC plot obtained from post processing in cryoSPARC v4.1. The dashed line represents a 0.143 cut-off used to define the overall nominal resolution of the map which corresponds to the corrected curve (in navy). For the curve labelled as masked, the FSC was calculated using a mask with soft edges. **c)** Model validation showing in black the 3DFlex refinement [3] improvement (negative values of loss) compared to the unmodified map (non-3DFlex map) throughout the training iterations and in blue the change of penalty measured relative to the unmodified map. Acceptable values should reach a plateau signal below 1. **d)** Latent distribution 2D-plots of the dimensions (eigen vectors) used to describe the conformational heterogeneity in the batch of particles. The 3DFlex refinement is considered valid if the values are within and not too close to the ± 1.5 range.

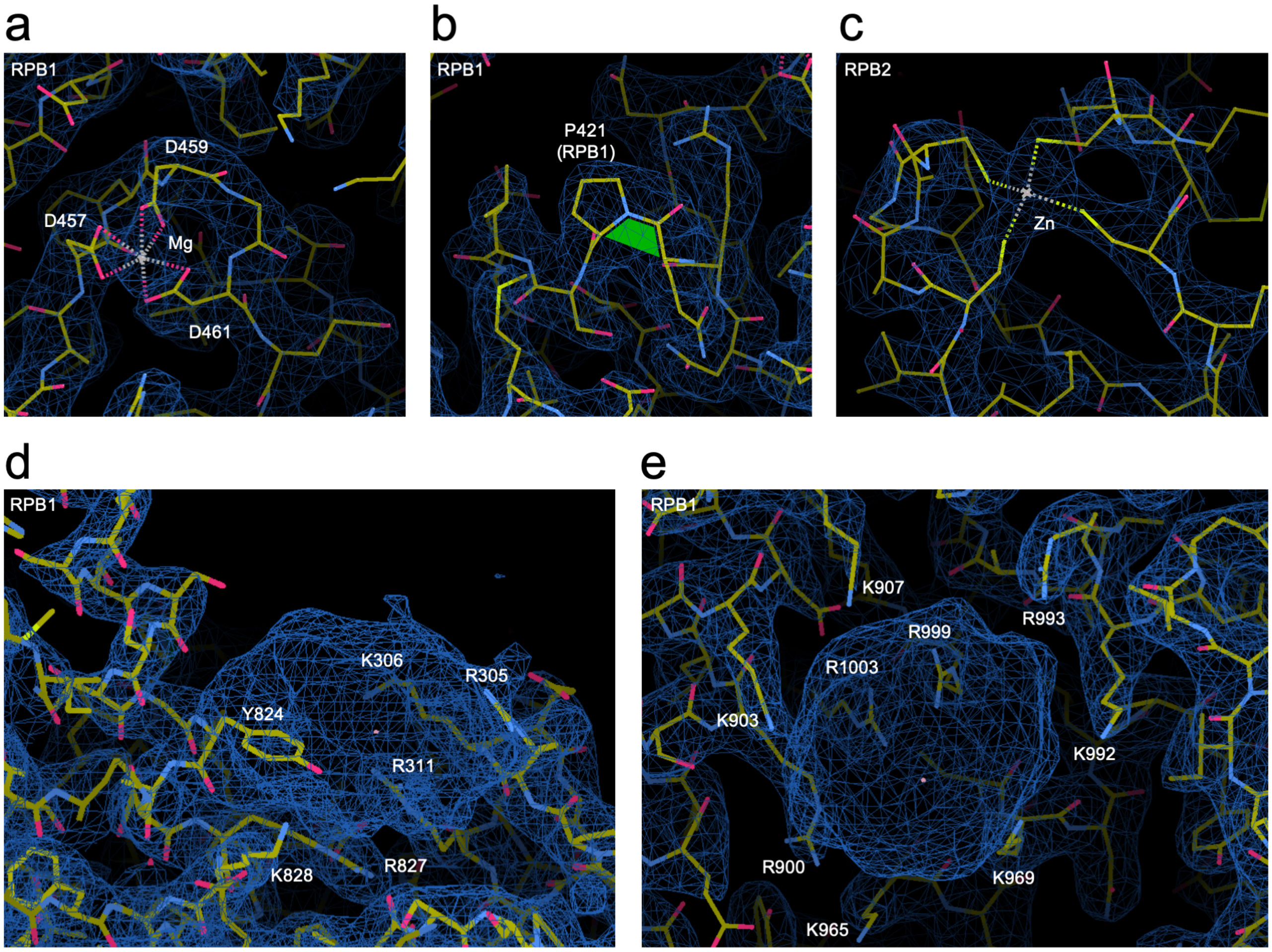

Supplementary Figure 5. Details of the ASFV RNAP model refined inside the EM map. a) The catalytic site of the RNA polymerase showing the aspartate triad fully coordinated with the Mg ion at the centre. **b)** The ASFV RNAP contains two proline residues in the cis configuration in position 373 and 421 (shown here) of vRPB1. **c)** vRPB2 zinc finger domain. This is one of the six zinc finger domains found in the EM map. **d-e)** The two extra-densities are shown next to the bridge helix in the DNA-binding channel (**d**) and bound to the foot domain of RPB1 (**e**). All residues involved in the binding of these two unidentified ligands are labelled. In all panels the map is shown as a blue net with the structure in sticks modelled inside. The carbon chains are coloured in gold, oxygen in red, nitrogen in blue, and sulphur in yellow. The ions are depicted as white crosses while their coordination systems are rendered by dashed lines. The cis configuration of P421 is highlighted as a green polygon. Figure prepared in Coot v0.8.9 [4].

vRPB1

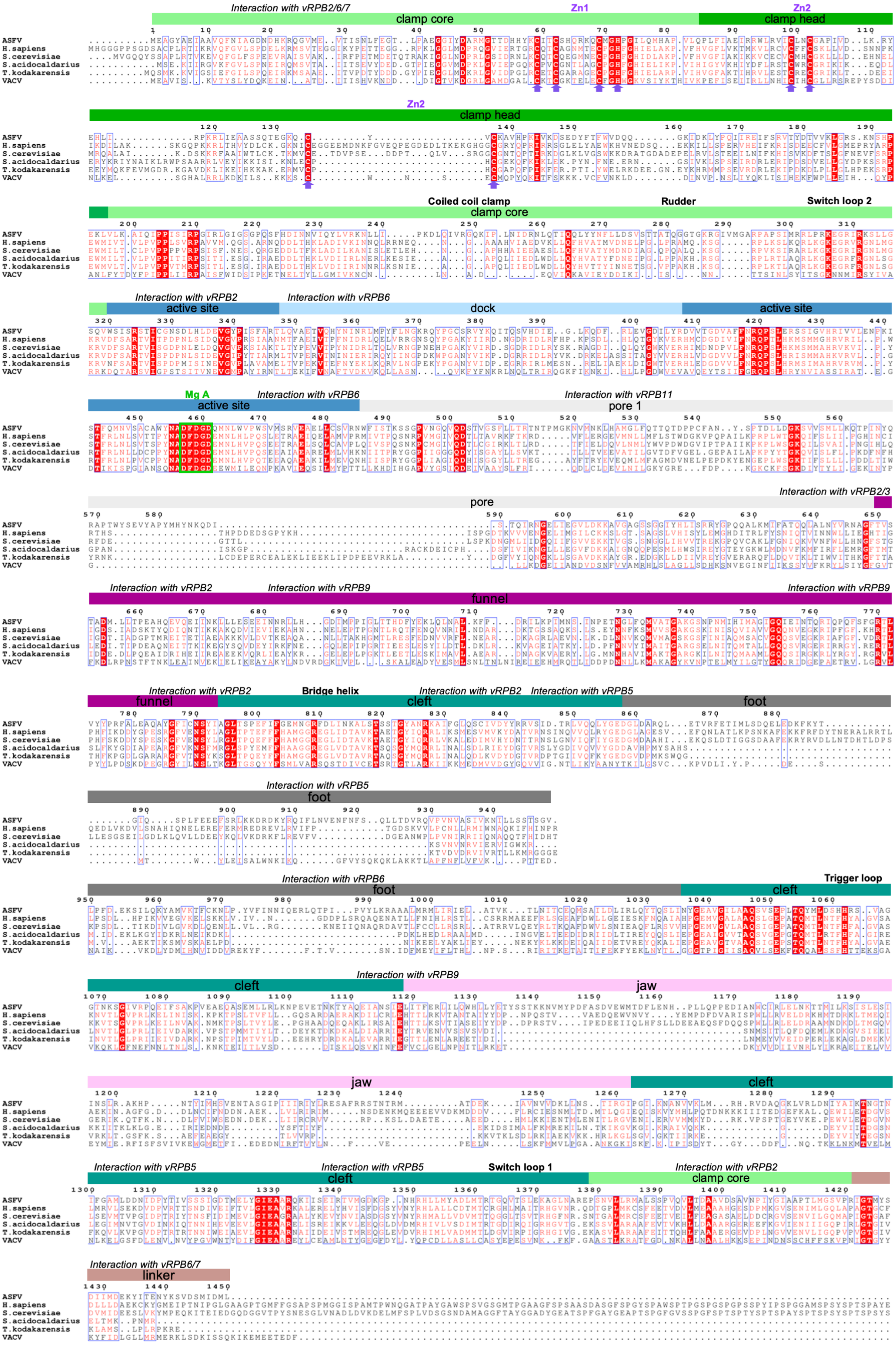
Interaction with vRPB2/6/7
clamp core
Zn1
Zn2
clamp head
ASFV
H.sapiens
S.cerevisiae
S.acidocaldarius
T.kodakarensis
VACV
Coiled coil clamp
Rudder
Switch loop 2
Interaction with vRPB2
Interaction with vRPB6
active site
dock
Mg A
Interaction with vRPB11
pore 1
pore
Interaction with vRPB2/3
Interaction with vRPB9
funnel
Bridge helix
cleft
Interaction with vRPB5
foot
Trigger loop
jaw
Switch loop 1
Interaction with vRPB6/7
linker

vRPB2

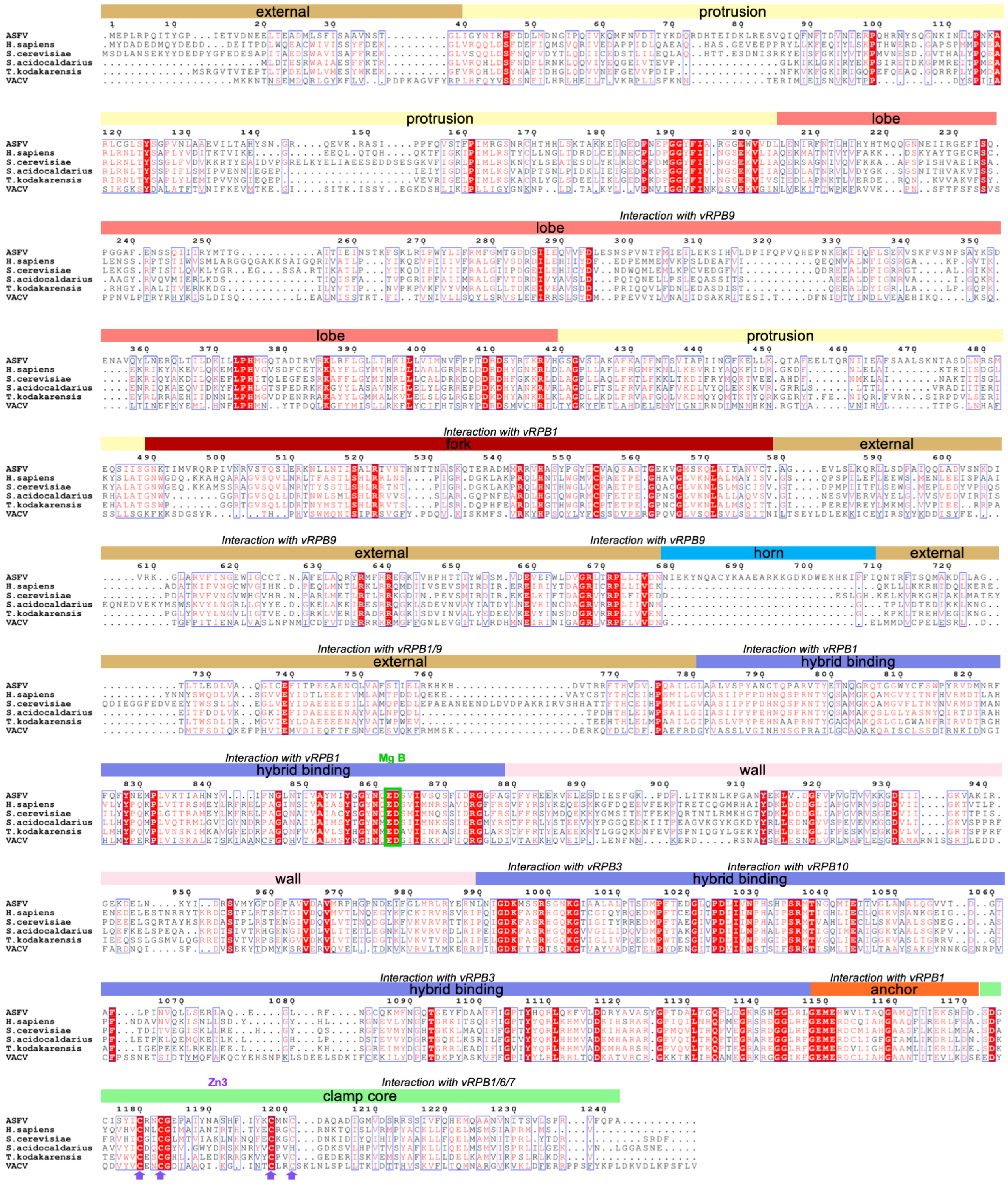

Supplementary Figure 6. Structure-based sequence alignment of the vRPB1 and vRPB2 catalytic subunits. The sequences of RPB1 and RPB2 from five different species of eukaryotes, archaea, and VACV have been aligned to the ASFV subunits and visualized using the Espript 3 [5] webserver. The residues are highlighted in gradient of red, based on conservation, applying a threshold of 0.6 (in a range from 0 to 1). The structures used for the structure-based MSA are *Homo sapiens* (pdb 5iyc [6]), *Saccharomyces cerevisiae* (pdb 7o75 [7]), *Sulfolobus acidocaldarius* (pdb 7ok0 [8]), *Thermococcus kodakarensis* (pdb 6kf3 [9]), and *Vaccinia* virus (pdb 7amv [10]). On the top of each MSA a schematic of the ASFV vRPB1 and vRPB2 domain organization has been added, highlighting the position of zinc fingers (arrows in medium purple), the catalytic Mg binding sites (green box), specific motifs, and the interfaces with other RNAP subunits. The domains have been assigned according to Cramer et al., 2001 [11] with the exception of the horn which is an ASFV-specific motif. vRPB1 MSA was split in two in order to follow the archaeal subunit composition where the Rpo1 subunit is split in Rpo1N and Rpo1C along the foot domain.

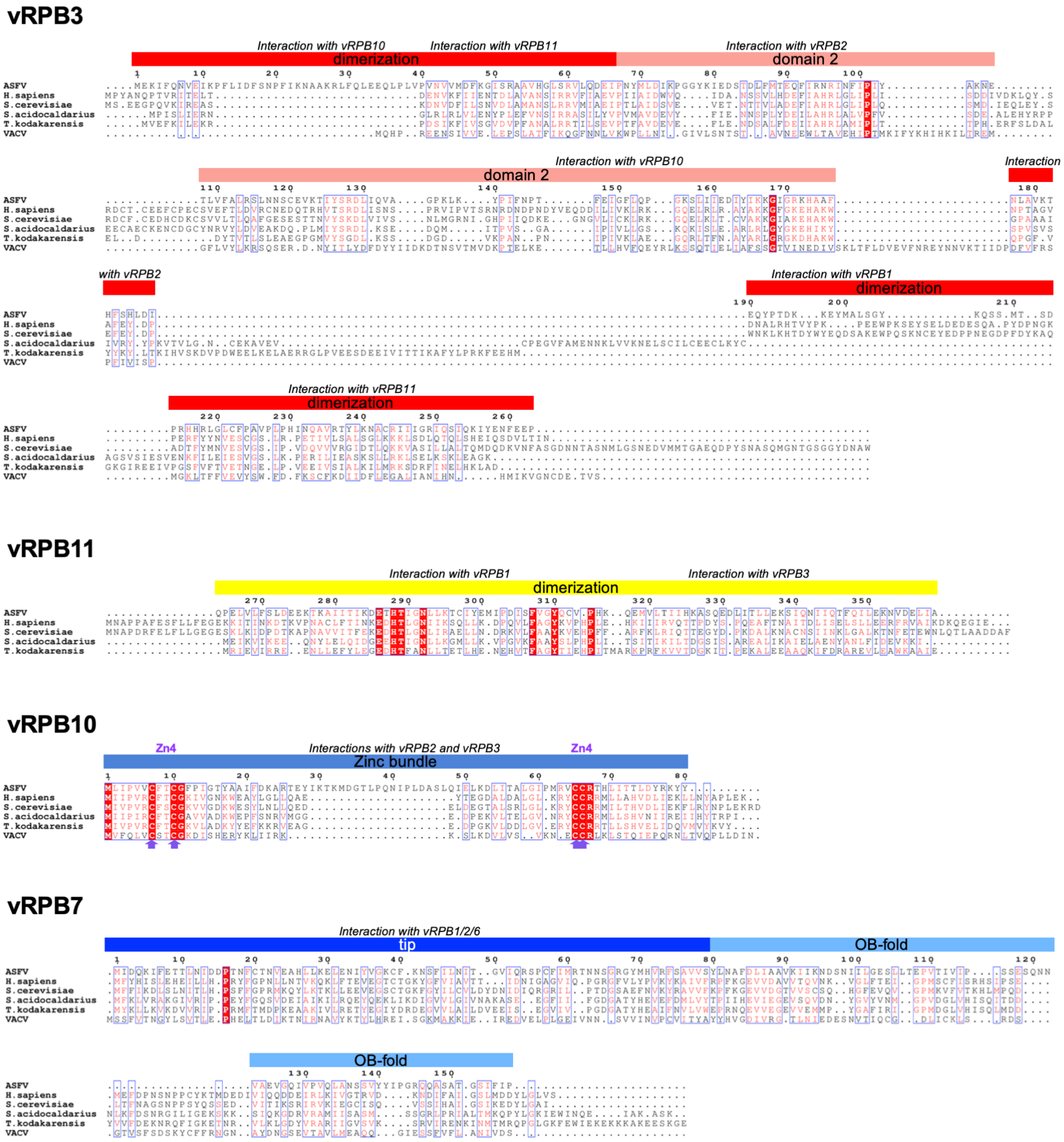
vRPB3
Interaction with vRPB10
Interaction with vRPB11
Interaction with vRPB2
dimerization
domain 2
Interaction
with vRPB2
Interaction with vRPB1
ASFV
H.sapiens
S.cerevisiae
S.acidocaldarius
T.kodakarensis
VACV
vRPB11
Interaction with vRPB1
Interaction with vRPB3
vRPB10
Zn4
Interactions with vRPB2 and vRPB3
Zinc bundle
vRPB7
Interaction with vRPB1/2/6
tip
OB-fold

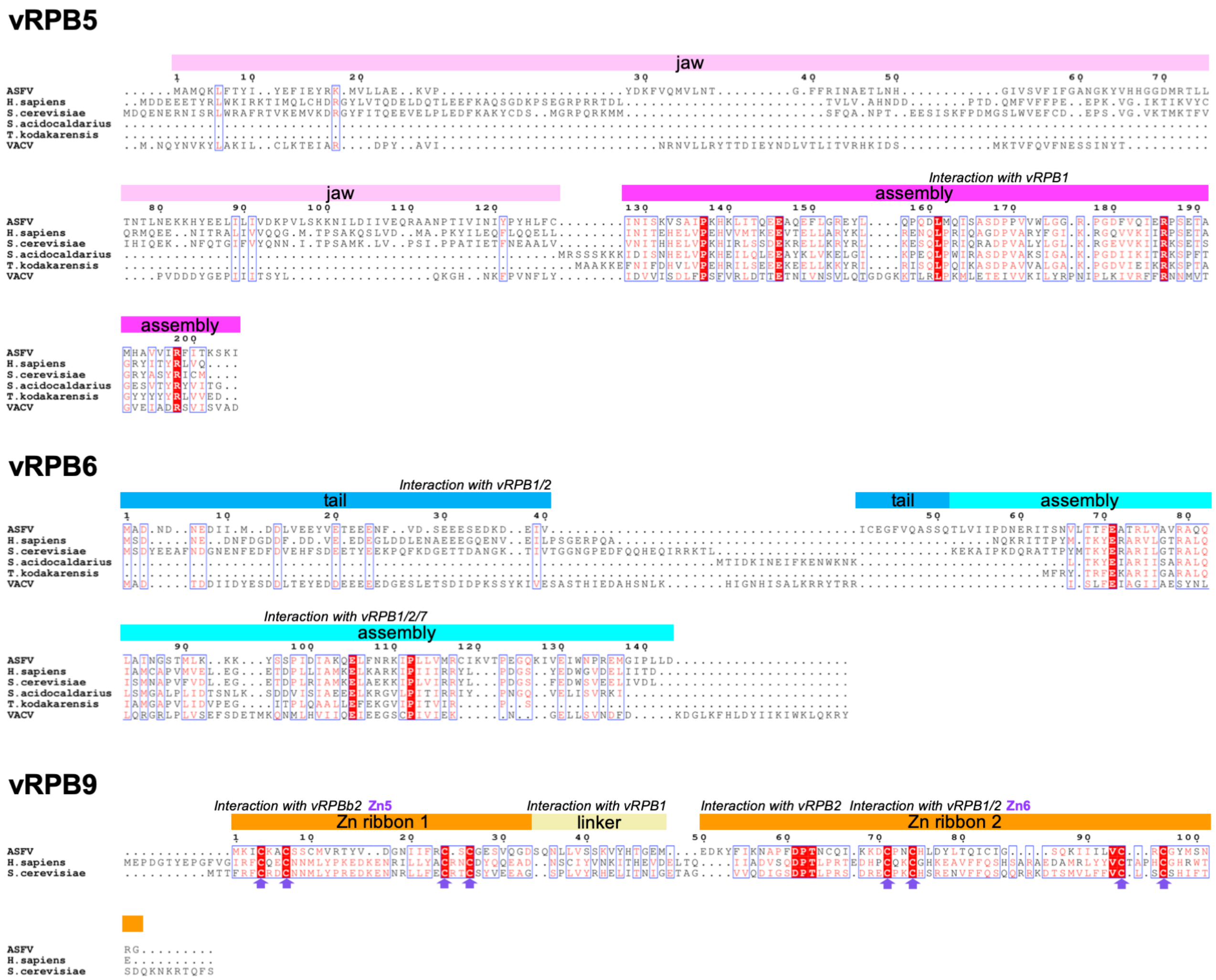

Supplementary Figure 7. Structure-based sequence alignment of the assembly and auxiliary subunits. The sequences of ASFV vRPB3-11, vRPB10, vRPB7, vRPB5, vRPB6, and vRPB9 have been aligned to the corresponding homologs from eukaryotes, archaea, and VACV, and visualized using the Espript 3 webserver. Residues are highlighted in gradient of red based on conservation applying a threshold of 0.6 (in a range from 0 to 1). Structures used for the alignment are *H. sapiens* (pdb 5iyc), *S. cerevisiae* (pdb 7o75), *S. acidocaldarius* (pdb 7ok0, and 7oqy [8] for RPO7), *T. kodakarensis* (pdb 6kf3), and VACV (pdb 7amv). On the top of the MSA a schematic of the domain organization has been added, highlighting the position of the zinc finger (arrows in medium purple), and the interfaces with other RNAP subunits. The domains have been assigned according to Cramer et al., 2001 and Fernandez-Tornero et al., 2013 [12]. For clarity, RPB3-11 of both ASFV and VACV has been split to allow the alignment with the correct subunit. However, VACV RPB11 homolog has been removed from the sequence alignment because it is not conserved either at sequence, nor structural level, although occupying a similar position. Similarly, the C-terminal domain (CTD) of ASFV RPB7 has been removed because it is absent in other domains of life and with no sequence homology with other characterised proteins. RPB9 is not present in archaea or VACV, as well as RPB8 and 12 are not present in either ASFV and VACV.

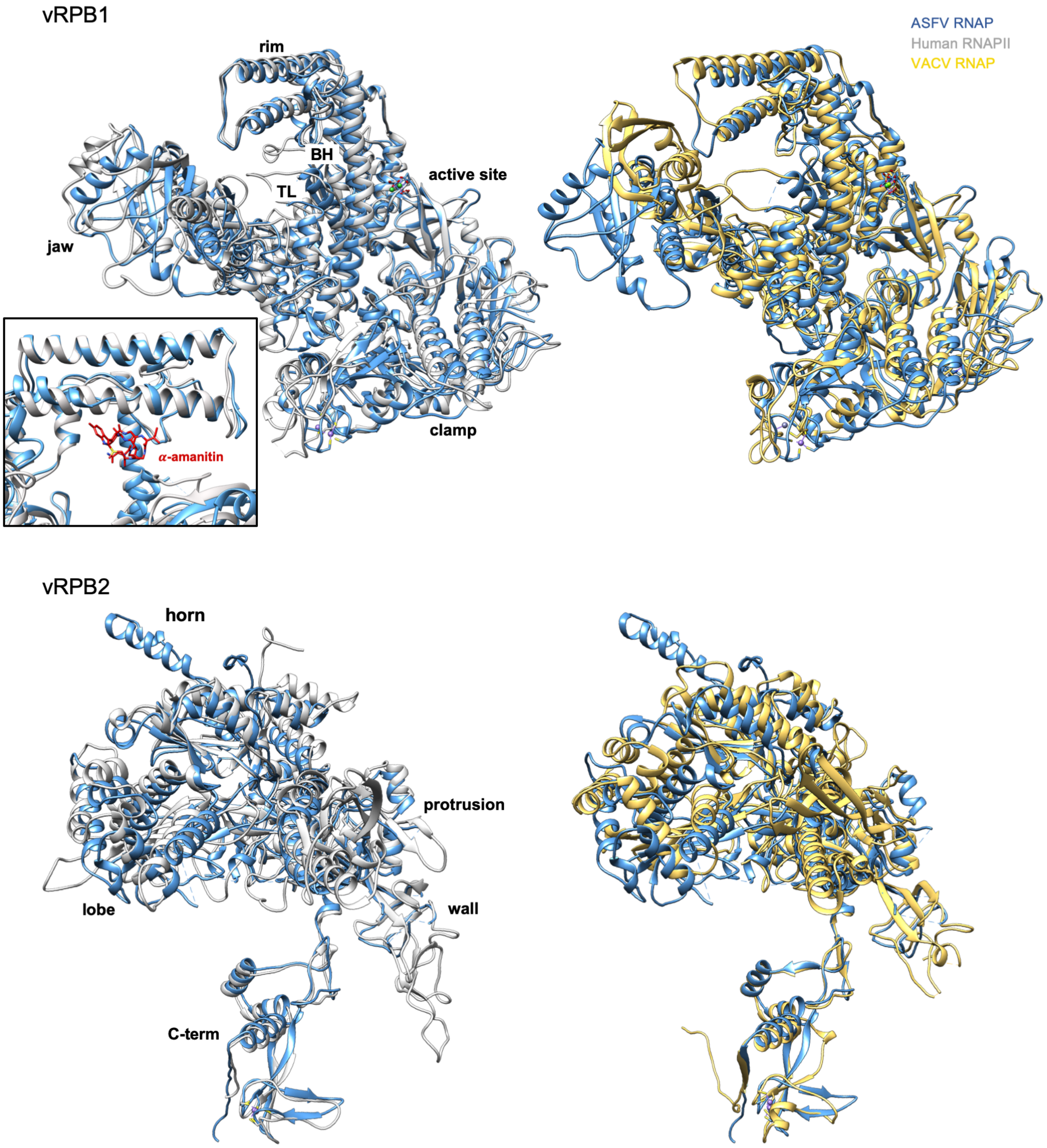

Supplementary Figure 8. Structure comparison of the ASFV core subunits RPB1 and RPB2 with human and VACV. Ribbon style representation of vRPB1 and vRPB2, in cornflower blue for ASFV, light grey for human (pdb 5iyc), and yellow for VACV (pdb 7amv). Zinc ions are shown in medium purple and magnesium in green within their coordination systems, shown in sticks (yellow for sulphur, red for oxygen, and blue for nitrogen). The main domains and structural features are labelled. Enlargement of the alpha-amanitin binding site on the rim. Alpha-amanitin is shown in red sticks bound to RNAPII in grey (pdb code 3cqz [13]). Superpositions were carried out in Chimera.

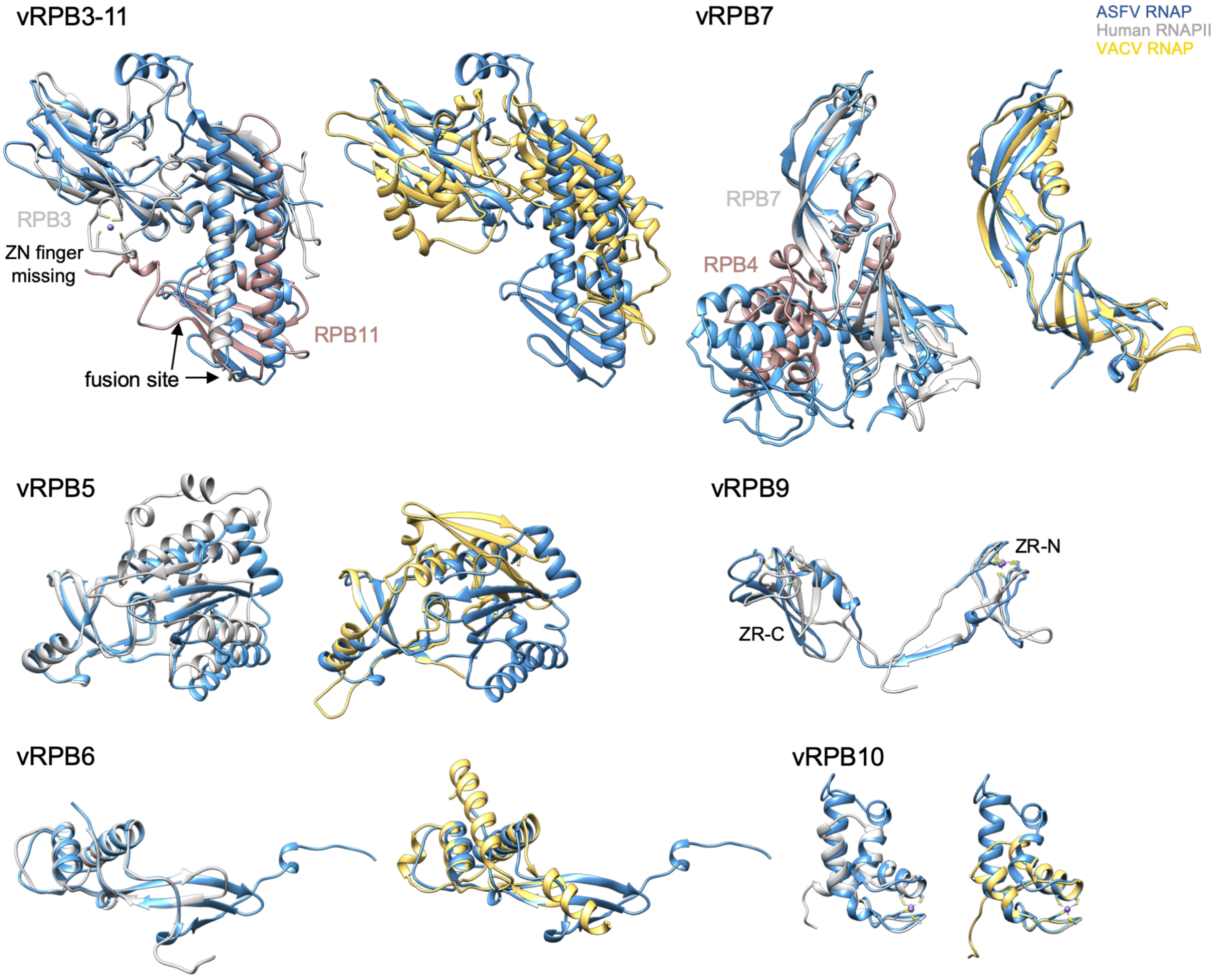

Supplementary Figure 9. Structure comparison of the ASFV small subunits with human and VACV. Ribbon style representation for each subunit, in cornflower blue for ASFV, light grey for human (pdb 5iyc), with the exception of RPB11 and RPB4 highlighted in rosy brown, and yellow for VACV (pdb 7amv). Zinc ions are shown in medium purple within their coordination systems, shown in sticks (yellow for sulphur). The main domains and structural features are indicated. RPB9 and RPB4 are missing in VACV, while the CTD of ASFV vRPB7 and VACV RPB11 are not homologues of human RPB4 and RPB11, respectively, despite occupying the same positions within the structures of ASFV and VACV RNAP. RPB8 and 12 are absent in ASFV and VACV. Superpositions were performed in Chimera.

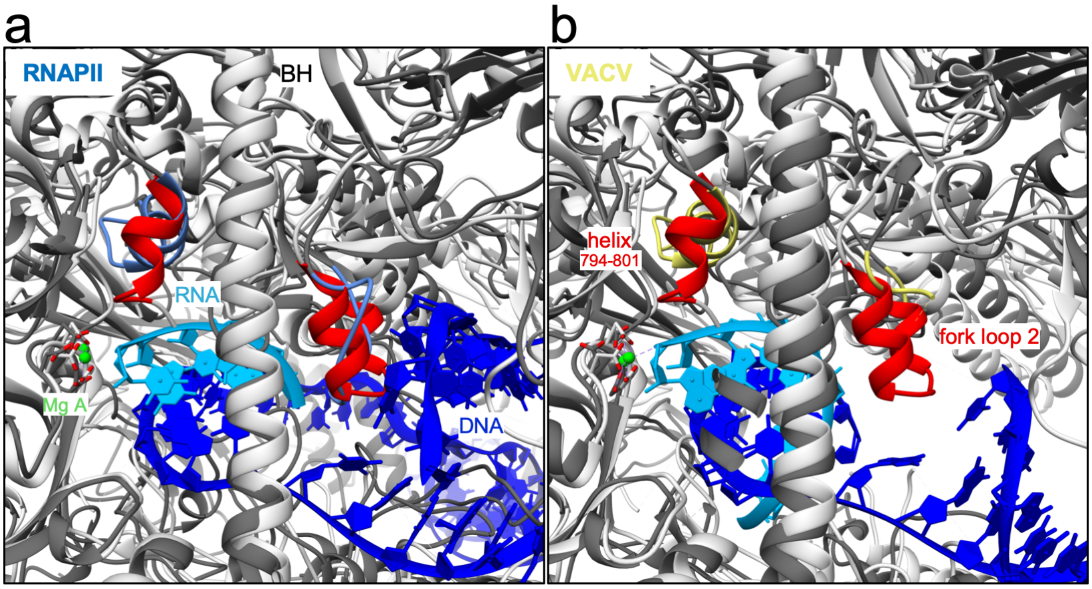

Supplementary Figure 10. Superposition of the ASFV closed conformation with the human RNAPII and VACV RNAP initially transcribing structures. The catalytic core of ASFV RNAP (in light grey ribbon) was superimposed with the **(a)** human RNAPII (pdb 5iyd [6], in dark grey) and **(b)** VACV RNAP (pdb 7aoh [10], in dark grey) using vRPB2 in Chimera. The most relevant differences, vRPB2 helix 794-801 and the fork loop 2, are highlighted in red for ASFV, while the homologous structural features in RNAPII and VACV RNAP are coloured in cornflower blue and in pale yellow, respectively. DNA is shown in blue, RNA in sky blue, the catalytic magnesium in green. BH stands for bridge helix.

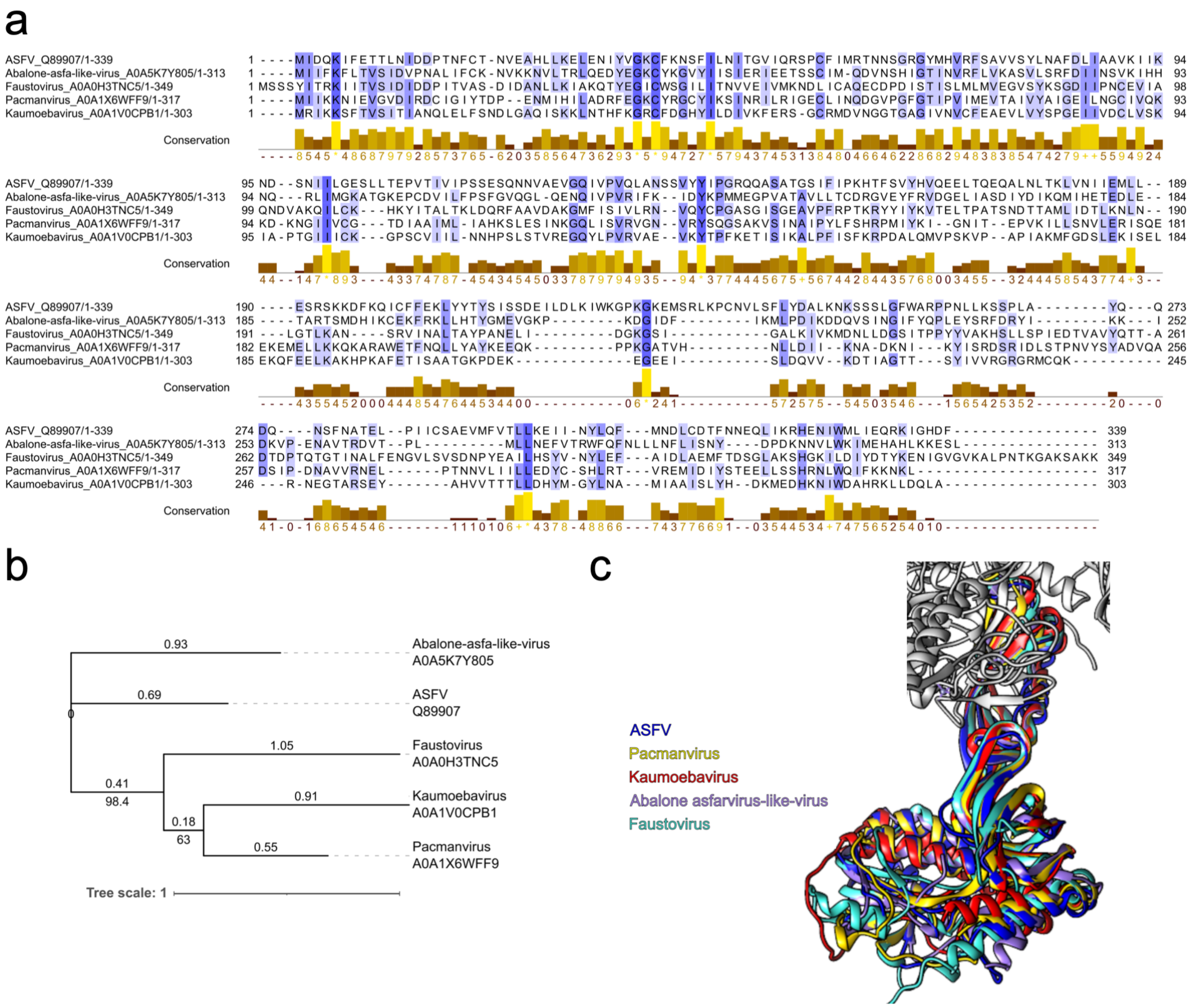

Supplementary Figure 11. Sequence conservation of the ASFV RPB7 CTD in other NCLDVs. a) Clustal Omega MSA [14] of putative homologs in other NCLDVs for ASFV vRPB7 displayed using Jalview [15]. These matches were identified using PSI-BLAST [16] (default settings) of the BA71V strain vRPB7 sequence. Their Uniprot identifiers are listed to the left of the MSA. **b)** Phylogenetic tree generated using IQ-TREE [17] (v1.6.12) from the MSA with 100 non-parametric bootstrap replicates. Length is shown above branches, bootstrap values below branches, and the tree was visualised using iTOL (v6.7.4) [18]. **c)** Superimposition of ASFV vRPB7 from our structure with AlphaFold2-predicted models of homologs from other viruses reported in panel **(a)**, carried out using Chimera Matchmaker (default settings) [19]. The vRPB7 NTD and CTD domains of Faustovirus and Abalone asf-like virus models were split to facilitate structural alignment. AlphaFold2 modelling of viral homologs utilised ColabFold (v1.5.2) [20] AlphaFold2 [21] using MMseqs2. Settings were as default, besides num_relax set to 1 and template_mode as pdb70.

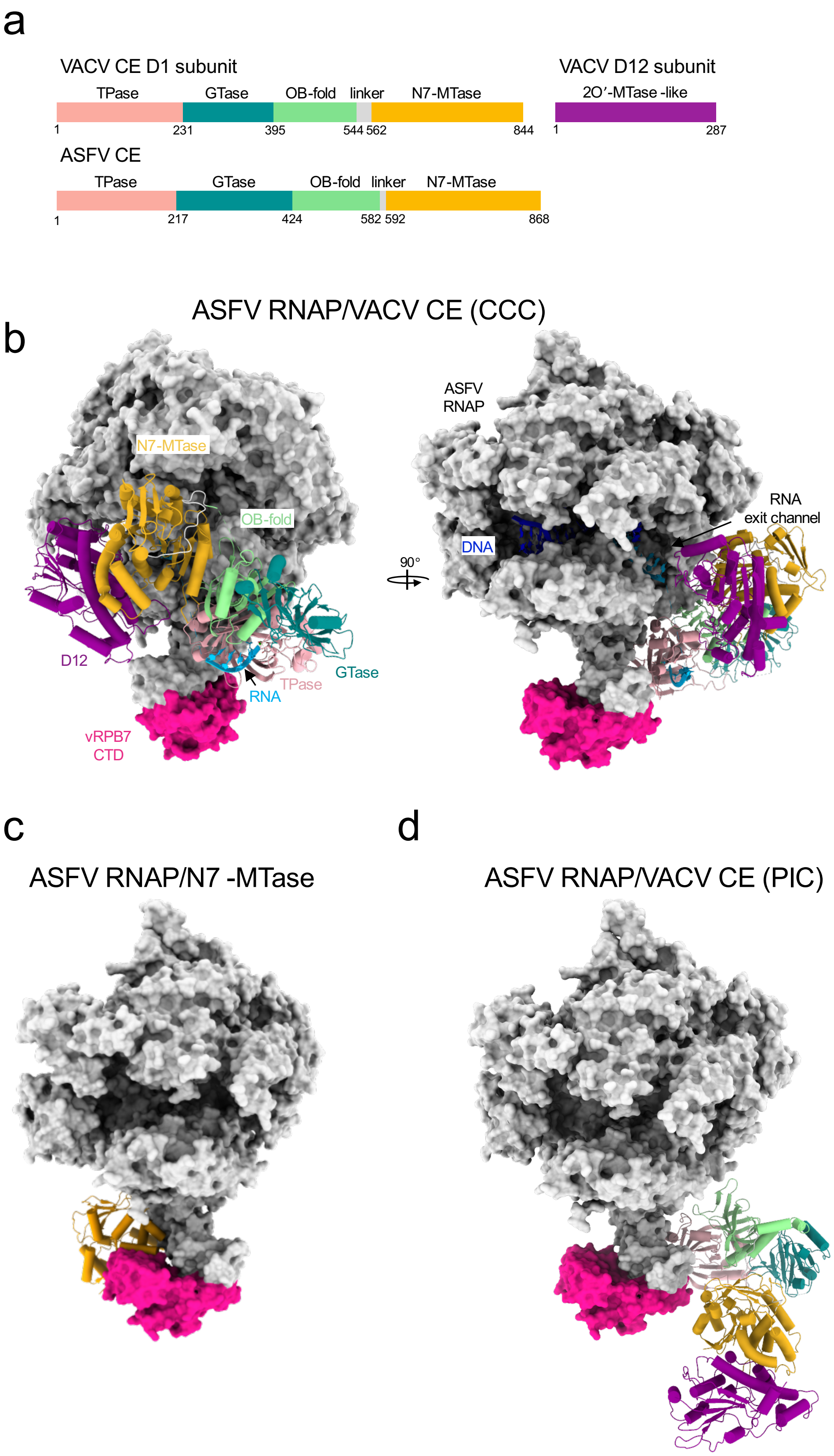

Supplementary Figure 12. Generation of the ASFV RNAP/CE complex models. a) Domain organization for the capping enzymes (CE) in VACV and ASFV. **b)** The VACV CCC structure (co-transcriptional capping complex, pdb 6rie [22]) was superimposed to the ASFV closed conformation shown as surface in grey with the vRPB7 CTD highlighted in deep pink. From the superposition only the VACV CE and the DNA/RNA scaffold are shown in ribbon style and the model is presented in two orientations. The scaffold is shown in blue for the DNA and sky blue for the RNA. The VACV CE is coloured according to the domain organization. For the D1 subunit, the triphosphatase (TPase) domain is in pink, the guanylyltransfarase (GTase) in teal, the OB-fold domain in light green, and the N7-methyltransferase (N7-MTase) in dark gold. The D12 subunit (2´O-MTase-like domain) is shown in dark magenta. All the following panels were prepared maintaining the same colour coding. **c)** Model

of the ASFV RNAP/N7-MTase obtained by superimposing the human 2´O-MTase (pdb 4n48, chain B [23]) to the vRPB7 CTD domain of ASFV first, then superimposing the VACV D1 N7-MTase/D12 complex to the human 2´O-MTase. Finally, the crystal structure of the ASFV CE N7-MTase domain was superimposed to the VACV homologous domain. **d)** The VACV pre-initiation complex (PIC, pdb 6rfl [24]) was used for the superimposition. All superpositions were prepared in Chimera using vRPB2 as reference, while panels showing the vRNAP as surface were prepared in Chimera X [25].

Supplementary Tables

Supplementary Table 1. Mass spectrometry analysis

Uniprot accession code	Number of peptides	Protein Name	Species	ASFV new name
NP_042747.1	1805	RNA polymerase subunit 2	ASFV	vRPB2
NP_042792.1	1777	RNA polymerase subunit 1	ASFV	vRPB1
P0C9E7.1	1109	Putative DNA-directed RNA polymerase subunit 3 homolog	ASFV	vRPB3-11
sp|ALBU_HUMAN|	1081	Albumin	Human	
P60712	416	Actin, cytoplasmic 1	Bos taurus	
P0C9B4.1	310	Putative DNA-directed RNA polymerase subunit 5 homolog	ASFV	vRPB5
P0CAE9.1	280	Uncharacterized protein D339L	ASFV	vRPB7
P0C9D6.1	225	DNA-directed RNA polymerase subunit 6 homolog	ASFV	vRPB6
ENSEMBL:ENSBTAP00000016242	203	Similar to alpha-tubulin I isoform 1	Bos taurus	
NP_042788.1	201	RNA polymerase subunit 10	ASFV	vRPB10
QED21635.1	173	C122R	ASFV	vRPB9
AWW06428.1	416	DNA polymerase, partial	Uncultured cyanophage	
First 12 hits obtained from the mass spectrometry analysis after Trypsin treatment. Obvious contaminants like human keratin and Trypsin have been removed from the list.				

Supplementary Table 2. RPB7 CTD DALI structural homology search main results.

Molecule name	Z-score	rmsd	lali	nres	%id	PDB
Homo sapiens Cap-specific mRNA (nucleoside-2'-O-)-methyltransferase 1	6.3	2.8	127	406	8	4n48-B
Rattus norvegicus Signal transducer and activator of transcription	6.2	7.3	65	128	8	7zn7-D
R. norvegicus Synaptosomal-associated protein 25	6.1	5.0	68	147	9	6mdm-H
Polytomella sp. Asa-10: Polytomella F-ATP synthase associated protein ASA1	6.1	5.4	89	595	7	6rd4-1
Bacillus sp. Nicking endonuclease N.BspD6I	5.9	4.0	86	594	8	4wl5-A
Escherichia coli Mechanosensitive channel MscK	5.8	5.5	71	723	11	7uw5-A
Saccharomyces cerevisiae Transcriptional activator SPT7	5.8	5.2	62	279	13	6t9i-K
Wolinella succinogenes MCCA	5.7	3.3	75	660	4	4rkm-B
S. cerevisiae Separin	5.7	9.0	76	1488	5	5u1s-A
Methanothermobacter thermautotrophicus Uranyl-binding protein	5.6	4.6	64	82	6	4fzo-A
Schizosaccharomyces pombe Switch-activating protein 1	5.6	7.4	70	110	6	5b7j-A
Streptococcus parasanguinis Fimbriae-associated protein Fap1	5.5	3.3	73	88	14	3rgu-A
Nakaseomyces glabratus Mediator of RNA Polymerase II Transcription Subunit 15	5.5	3.6	72	86	13	4d7x-A
H. sapiens Nucleoporin NUP188 homolog	5.4	5.5	75	1256	8	5ijo-J
Legionella pneumophila Lem22	5.3	7.5	64	90	14	5wd8-A
E. coli Sensor histidine kinase EnvZ	5.3	8.0	61	222	7	4cti-D
H. sapiens STAGA complex 65 subunit gamma	5.3	9.9	68	219	9	7ktr-D
Streptococcus suis antigen HP0197	5.3	3.4	87	154	9	4fz4-A
Vaccinia virus Small subunit of mRNA capping enzyme Rpo147	5.3	3.1	116	287	3	6rie-L
Staphylococcus aureus Septation ring formation regulator EZRA	5.3	3.2	67	194	3	4uy3-A
S. cerevisiae Non-structural maintenance of chromosome element 4	5.3	7.0	67	307	7	7tve-G
Desulfovibrio vulgaris Hydroxylamine reductase	5.2	2.7	74	553	9	1e1d-A
Sinorhizobium meliloti Putative polyphosphate kinase 2	5.2	4.3	76	288	8	3czq-A
Listeria monocytogenes anti-CRISPR protein AcrIIA1	5.2	3.0	61	147	7	5y69-A
Vibrio cholerae Antitoxin protein TsiV3	5.2	3.7	74	95	9	4noo-B
H. sapiens cGAS-like receptor protein MB21D2	5.2	4.2	92	403	9	7lt1-A
Drosophila melanogaster Protein timeless	5.2	3.8	65	759	9	8dd7-B
Streptococcus pneumoniae Putative transcriptional regulator PezA	5.2	3.2	73	95	7	2p5t-E
H. sapiens Centromere/kinetochore protein zw10 homolog	5.1	9.2	80	779	13	7qpg-W
Thermotoga maritima Phosphate transport system accessory protein PhoU homolog 2	5.1	6.7	74	224	9	1sum-B
Leptolyngbya sp. Dual sensor histidine kinase PPHK	5.1	8.2	66	305	8	6oap-A
Corynebacterium glutamicum Transcriptional regulator	5.1	9.0	72	188	14	2o7t-A
H. sapiens Dedicator of cytokinesis protein 5	5.1	9.9	77	1642	6	7dpa-A
Haemophilus influenzae Uncharacterized tRNA/rRNA methyltransferase HI038	5.1	3.9	60	239	15	3ilk-A
Archaeoglobus fulgidus AF2299 Protein, *Renibacterium salmoninarum* Phosphatidylinositol Synthase (chimera)	5.1	2.8	68	342	7	5d92-B
H. sapiens Activating signal cointegrator 1 complex subunit 2	5.0	9.3	86	396	7	6yxq-B
Streptococcus pyogenes Antitoxin epsilon	5.0	3.5	72	87	6	1gvn-A
Thermochaetoides thermophila Nucleoporin NUP192	5.0	7.2	63	1542	5	7mvv-A
Yarrowia lipolytica Subunit NUFM of NADH-Ubiquinone oxidoreductase (Complex I)	5.0	3.6	72	126	11	6yj4-V
H. sapiens LYR motif-containing protein 4, mitochondrial desulfurase	5.0	3.2	69	85	4	6uxe-B
Geobacter sulfurreducens CRISPR-associated exonuclease Cas4/endonuclease Cas1 fusion	5.0	8.5	83	554	5	7mi4-A

The pdb structure obtained after model refinement of the RPB7 CTD domain was used to carry out the structural homology search using the DALI webserver [26] against the non-redundant structures database PDB25. The results were trimmed by removing all structures belonging to membrane proteins and synthetic constructs, both not relevant in this study, or with a Z-score below 5.

Supplementary Table 3. Primers used for ASFV RNAP cloning

Primer Name	Sequence	Description
pLIB_amp_FW	GACAAGCTTGTCGAGAAGTACTAGAGG	Linearise pLIB via PCR for assembly with all subunits
pLIB_amp_RV	CGTCGACGTAGGCCTTTGAATTCC	Linearise pLIB via PCR for assembly with all subunits
GA_pLIB+RPB1_FW	CAAAGGCCTACGTCGACGATGGAGGCTGGATATGC	Amplify RPB1 to assemble with linear pLIB
GA_pLIB+RPB1_RV	CCTCTAGTACTTCTCGACAAGCTTGTCCTATAACATGTCAATCATGG	Amplify RPB1 to assemble with linear pLIB
GA_ZZ_into_pLIB_FW	GAATTCAAAGGCCTACGTCGACGATGTCTTACTATCACCACCACC	Amplify zz-tag to assemble with linear pLIB
GA_ZZ_into_pLIB_RV	TTCTCGACAAGCTTGTCACCCTGGAAATAGAGATTCTCTGTAGTTGG	Amplify zz-tag to assemble with linear pLIB
GA_pLIB+ZZ_FW	GGAATTCAAAGGCCTACGTCGACGATGTCTTACTATCACCACC	Amplify RPB2 to assemble with pLIB:zz
GA_pLIB+RPB2_RV	CCTCTAGTACTTCTCGACAAGCTTGTCTTAAGCCGGTTGAAATACTCTTGG	Amplify RPB2 to assemble with pLIB:zz
GA_pLIBzz+2_AMP_FW	CCAAGAGTATTTCAACCGGCTTAAGACAAGCTTGTCGAGAAGTACTAGAGG	Amplify pLIB:zz to linearise and assemble with RPB2
GA_pLIB+TEV-zz_RV	CCGCAAGGGTTCCATACCCTGGAAATAGAGATTCTC	Amplify pLIB:zz to linearise and assemble with RPB2
GA_pLIB+RPB3_FW	GAATTCAAAGGCCTACGTCGACGATGGAAAAAATTTTCCAAAACG	Amplify RPB3 to assemble with linear pLIB
GA_pLIB_RPB3_RV	CTAGTACTTCTCGACAAGCTTGTCTTAAGCAATCAGTTCATCAACATTTTTTTCAAGG	Amplify RPB3 to assemble with linear pLIB
GA_pLIB_RPB5_FW	GAATTCAAAGGCCTACGTCGACGATGGCCATGCAAAAGTTATTTACGTATATTTACG	Amplify RPB5 to assemble with linear pLIB
GA_pLIB+RPB5_RV	CCTCTAGTACTTCTCGACAAGCTTGTCTCAAATTTTGGACTTGG	Amplify RPB5 to assemble with linear pLIB
GA_pLIB+RPB6_FW	GGAATTCAAAGGCCTACGTCGACGATGGCTGATAATGACAACGAGG	Amplify RPB6 to assemble with linear pLIB
GA_pLIB+RPB6_RV	CCTCTAGTACTTCTCGACAAGCTTGTCCTAGTCTAACAGCGGGATGCC	Amplify RPB6 to assemble with linear pLIB
GA_pLIB+RPB7_FW	GGCCTACGTCGACGATGATCGACCAAAAAATTTTTGAGACAACG	Amplify RPB7 to assemble with linear pLIB
GA_pLIB+RPB7_RV	CCTCTAGTACTTCTCGACAAGCTTGTCTTAAAAATCATGTCC	Amplify RPB7 to assemble with linear pLIB
GA_pLIB+RPB9_FW	GGAATTCAAAGGCCTACGTCGACGATGAAAATTTGTAAGG	Amplify RPB9 to assemble with linear pLIB
GA_pLIB+RPB9_RV	CTTCTCGACAAGCTTGTCTTATCCTCTGTTGCTCATATAGC	Amplify RPB9 to assemble with linear pLIB
GA_pLIB+RPB10_FW	GGAATTCAAAGGCCTACGTCGACGATGTTGATACCGGTGG	Amplify RPB10 to assemble with linear pLIB
GA_pLIB+RPB10_RV	GCTGATTATGATCCTCTAGTACTTCTCGACAAGCTTGTCTTAATAATATTTACGATAATCC	Amplify RPB10 to assemble with linear pLIB
GAαR	AAATCTTTAGACCATAGAGCGTTCAC	Stepwise assembly of pBIG1
DampF	AAACCTAATGATGCCTGATGTTTC	To PCR amplify pBIG2abc, replacing the ‘A’ overlap sequence for the ‘B’ sequence – generating a pBIG2bc vector for Gibson assembly.
GAAtoBR	AAACCCCGATTGAGATATAGTTTAATTAACCTAGGCCAGATAAC	
GADtoBF	AAACTATATCTCAATCGGGGTTTAAACCTAATGATGCCTGATGTTTTTATC	To PCR amplify fragment ‘D-5-7-E’, replacing the ‘D’ overlap sequence for the ‘B’ sequence – generating fragment ‘B-5-7-E’ for Gibson assembly with ‘A-6-1-B’ using the B overlap.
EampR	AAACTAAGCTATGTGAACCGTTTTTAATTAAC	

Supplementary References

1. Punjani, A., Rubinstein, J.L., Fleet, D.J. & Brubaker, M.A. cryoSPARC: algorithms for rapid unsupervised cryo-EM structure determination. *Nat Methods* **14**, 290-296 (2017).
2. Kimanius, D., Dong, L., Sharov, G., Nakane, T. & Scheres, S.H.W. New tools for automated cryo-EM single-particle analysis in RELION-4.0. *Biochem J* **478**, 4169-4185 (2021).
3. Punjani, A. & Fleet, D.J. 3DFlex: determining structure and motion of flexible proteins from cryo-EM. *Nat Methods* **20**, 860-870 (2023).
4. Casanal, A., Lohkamp, B. & Emsley, P. Current developments in Coot for macromolecular model building of Electron Cryo-microscopy and Crystallographic Data. *Protein Sci* **29**, 1069-1078 (2020).
5. Robert, X. & Gouet, P. Deciphering key features in protein structures with the new ENDscript server. *Nucleic Acids Res* **42**, W320-4 (2014).
6. He, Y. et al. Near-atomic resolution visualization of human transcription promoter opening. *Nature* **533**, 359-65 (2016).
7. Schilbach, S., Aibara, S., Dienemann, C., Grabbe, F. & Cramer, P. Structure of RNA polymerase II pre-initiation complex at 2.9 A defines initial DNA opening. *Cell* **184**, 4064-4072 e28 (2021).
8. Pilotto, S. et al. Structural basis of RNA polymerase inhibition by viral and host factors. *Nat Commun* **12**, 5523 (2021).
9. Jun, S.H. et al. Direct binding of TFEalpha opens DNA binding cleft of RNA polymerase. *Nat Commun* **11**, 6123 (2020).
10. Grimm, C., Bartuli, J., Boettcher, B., Szalay, A.A. & Fischer, U. Structural basis of the complete poxvirus transcription initiation process. *Nat Struct Mol Biol* **28**, 779-788 (2021).
11. Cramer, P., Bushnell, D.A. & Kornberg, R.D. Structural basis of transcription: RNA polymerase II at 2.8 angstrom resolution. *Science* **292**, 1863-76 (2001).
12. Fernandez-Tornero, C. et al. Crystal structure of the 14-subunit RNA polymerase I. *Nature* **502**, 644-9 (2013).
13. Kaplan, C.D., Larsson, K.M. & Kornberg, R.D. The RNA polymerase II trigger loop functions in substrate selection and is directly targeted by alpha-amanitin. *Mol Cell* **30**, 547-56 (2008).
14. Sievers, F. et al. Fast, scalable generation of high-quality protein multiple sequence alignments using Clustal Omega. *Mol Syst Biol* **7**, 539 (2011).
15. Waterhouse, A.M., Procter, J.B., Martin, D.M., Clamp, M. & Barton, G.J. Jalview Version 2--a multiple sequence alignment editor and analysis workbench. *Bioinformatics* **25**, 1189-91 (2009).
16. Altschul, S.F. et al. Gapped BLAST and PSI-BLAST: a new generation of protein database search programs. *Nucleic Acids Res* **25**, 3389-402 (1997).
17. Trifinopoulos, J., Nguyen, L.T., von Haeseler, A. & Minh, B.Q. W-IQ-TREE: a fast online phylogenetic tool for maximum likelihood analysis. *Nucleic Acids Res* **44**, W232-5 (2016).
18. Letunic, I. & Bork, P. Interactive Tree Of Life (iTOL) v5: an online tool for phylogenetic tree display and annotation. *Nucleic Acids Res* **49**, W293-W296 (2021).
19. Pettersen, E.F. et al. UCSF Chimera--a visualization system for exploratory research and analysis. *J Comput Chem* **25**, 1605-12 (2004).
20. Mirdita, M. et al. ColabFold: making protein folding accessible to all. *Nat Methods* **19**, 679-682 (2022).
21. Jumper, J. et al. Highly accurate protein structure prediction with AlphaFold. *Nature* **596**, 583-589 (2021).
22. Hillen, H.S. et al. Structural Basis of Poxvirus Transcription: Transcribing and Capping Vaccinia Complexes. *Cell* **179**, 1525-1536 e12 (2019).
23. Smietanski, M. et al. Structural analysis of human 2'-O-ribose methyltransferases involved in mRNA cap structure formation. *Nat Commun* **5**, 3004 (2014).
24. Grimm, C. et al. Structural Basis of Poxvirus Transcription: Vaccinia RNA Polymerase Complexes. *Cell* **179**, 1537-1550 e19 (2019).
25. Pettersen, E.F. et al. UCSF ChimeraX: Structure visualization for researchers, educators, and developers. *Protein Sci* **30**, 70-82 (2021).

26. Holm, L., Laiho, A., Toronen, P. & Salgado, M. DALI shines a light on remote homologs: One hundred discoveries. *Protein Sci* **32**, e4519 (2023).
