# Supplementary material for: Structure of the recombinant RNA polymerase from African Swine Fever Virus
Source: Nat Commun. 2024 Feb 21;15:1606. doi: 10.1038/s41467-024-45842-7 (PMC10881513; doi:10.1038/s41467-024-45842-7)
Supplement: Supplementary file 3 — Description of Additional Supplementary Files [file 41467_2024_45842_MOESM3_ESM.pdf]

Description of Additional Supplementary Files

File Name: Supplementary Movie 1
Description: ASFV RNAP DNA-binding channel opening. Particles were analysed using the 3D variability analysis (3DVA) and the extent of the DNA-binding channel widening was visualised in Chimera Volume series tool by using ten maps at approximately 5 Å resolution generated with the 3DVA Display tool.

File Name: Supplementary Movie 2
Description: ASFV RNAP shelf swivelling movement. The extent of the shelf-clamp swivelling obtained from 3DVA was visualised in Chimera Volume series tool by using ten maps at roughly 5 Å resolution generated with the 3DVA Display tool.

File Name: Supplementary Movie 3
Description: ASFV RNAP stalk flexibility. Particles were analysed by 3DVA and one of the most representative stalk movements (towards the clamp) was highlighted and visualised in Chimera Volume series tool by using ten maps at around 5 Å generated with the 3DVA Display tool.
