# Supplementary material for: Structure of the recombinant RNA polymerase from African Swine Fever Virus
Source: Nat Commun. 2024 Feb 21;15:1606. doi: 10.1038/s41467-024-45842-7 (PMC10881513; doi:10.1038/s41467-024-45842-7)
Supplement: Supplementary file 7 — Reporting Summary [file 41467_2024_45842_MOESM7_ESM.pdf]

nature portfolio

Corresponding author(s): Finn Werner

Last updated by author(s): Jan 19, 2024

Reporting Summary

Nature Portfolio wishes to improve the reproducibility of the work that we publish. This form provides structure for consistency and transparency in reporting. For further information on Nature Portfolio policies, see our Editorial Policies and the Editorial Policy Checklist.

Statistics

For all statistical analyses, confirm that the following items are present in the figure legend, table legend, main text, or Methods section.

n/a	Confirmed	
☐	☒	The exact sample size (n) for each experimental group/condition, given as a discrete number and unit of measurement
☐	☒	A statement on whether measurements were taken from distinct samples or whether the same sample was measured repeatedly
☒	☐	The statistical test(s) used AND whether they are one- or two-sided<br>*Only common tests should be described solely by name; describe more complex techniques in the Methods section.*
☒	☐	A description of all covariates tested
☒	☐	A description of any assumptions or corrections, such as tests of normality and adjustment for multiple comparisons
☐	☒	A full description of the statistical parameters including central tendency (e.g. means) or other basic estimates (e.g. regression coefficient) AND variation (e.g. standard deviation) or associated estimates of uncertainty (e.g. confidence intervals)
☒	☐	For null hypothesis testing, the test statistic (e.g. F, t, r) with confidence intervals, effect sizes, degrees of freedom and P value noted<br>*Give P values as exact values whenever suitable.*
☒	☐	For Bayesian analysis, information on the choice of priors and Markov chain Monte Carlo settings
☒	☐	For hierarchical and complex designs, identification of the appropriate level for tests and full reporting of outcomes
☒	☐	Estimates of effect sizes (e.g. Cohen's d, Pearson's r), indicating how they were calculated

Our web collection on statistics for biologists contains articles on many of the points above.

Software and code

Policy information about availability of computer code

Data collection	EPU v.2 software was used for EM data collection.<br>For the nonspecific in vitro transcription reactions , the beta emissions were measured using Tri-Carb2900TR with QuantaSmart v2.03 software (PerkinElmer) calibrated with industrial standards so that 1 Bq is equivalent to 60 CPM.

Data analysis

Relion v4.0 and cryoSPARC v.3 and v4.1 were used for data processing Phenix 1.20 was used for map sharpening; Phenix v1.20 and Coot v0.9.8.8 were used for model fitting and refinement. Molprobity webserver was used for model validation. Models of all RNAP subunits were generated with AlphaFold2 via 'AlphaFold Colab' available to use on Google Colab, with a Colab Pro account (https://colab.research.google.com/github/deepmind/alphafold/blob/main/notebooks/AlphaFold.ipynb).

Chimera v1.16 and ChimeraX v1.6.1 were used for macromolecule visualization, Esprit3 webserver for sequence alignment rendering.

The PDB25 database search within DALI webserver was used for the structural homology search.

For sequence alignment www.blast.ncbi.nlm.nih.gov/ was used after selecting blastp and PSI-BLAST options, and the alignment was visualised using Jalview v2.11.2.7. The phylogenetic tree was generated using IQ-TREE webserver (v1.6.12) and visualised using the iTOL webserver (v6.7.4).

AlphaFold2 modelling of viral paralogs of ASFV vRPB7 utilised ColabFold (v1.5.2) AlphaFold2, using MMseqs2. Settings were as default, besides num_relax set to 1 and template_mode as pdb70 (https://colab.research.google.com/github/sokrypton/ColabFold/blob/main/AlphaFold2.ipynb).

For the inhibitor testing, activity was calculated as the percentage of the mean average of the relevant positive controls. The mean average and standard deviation of test sample replicates was calculated using Microsoft Excel for Mac v16.77.1 software formulas AVERAGE and STDEV.

For manuscripts utilizing custom algorithms or software that are central to the research but not yet described in published literature, software must be made available to editors and reviewers. We strongly encourage code deposition in a community repository (e.g. GitHub). See the Nature Portfolio guidelines for submitting code & software for further information.

Data

Policy information about availability of data

All manuscripts must include a data availability statement. This statement should provide the following information, where applicable:

- Accession codes, unique identifiers, or web links for publicly available datasets
- A description of any restrictions on data availability
- For clinical datasets or third party data, please ensure that the statement adheres to our policy

Source data are provided with this paper as Source Data file. The data generated during the current study are available in the Protein Data Bank with the following PDB codes: 8Q3B [https://www.rcsb.org/structure/unreleased/8Q3B] for the closed conformation, and 8Q3K [https://www.rcsb.org/structure/unreleased/8Q3K] for the open conformation. The corresponding cryo-EM maps have been deposited on the Electron Microscopy Data Bank, with the following codes: EMD-18120 for the closed conformation, EMD-18129 for the composite map of the open conformation. In addition, we deposited the maps used to generate the composite map for the open conformation with the codes EMD-18163 for the consensus map, and EMD-18164 for the map of the stalk domain obtained from multibody refinement. The raw cryo-EM data are available upon request by writing to the corresponding author. The structures used for this study are: 7D8U [https://www.rcsb.org/structure/7D8U] (ASFV N7-MTase domain), 4N48 [https://www.rcsb.org/structure/4N48] (human 2´O-MTase), 6RIE [https://www.rcsb.org/structure/6RIE] (VACV CCC), 3K1F [https://www.rcsb.org/structure/3K1F] (yeast RNAPII/TFIIB), 6RFL [https://www.rcsb.org/structure/6RFL] (VACV inactive PIC), 5IYC [https://www.rcsb.org/structure/5IYC] (human PIC-OC, open complex), 7O75 [https://www.rcsb.org/structure/7O75] (yeast RNAPII PIC-OC), 7OK0 [https://www.rcsb.org/structure/7OK0] (S. acidocaldarius RNAP), 7OQY [https://www.rcsb.org/structure/7OQY] (S. acidocaldarius RNAP/TFS4), 6KF3 [https://www.rcsb.org/structure/6KF3] (T. kodakarensis RNAP) and 7AMV [https://www.rcsb.org/structure/7AMV] (VACV PIC-OC).

Research involving human participants, their data, or biological material

Policy information about studies with human participants or human data. See also policy information about sex, gender (identity/presentation), and sexual orientation and race, ethnicity and racism.

Reporting on sex and gender	Not applicable as no human participants are involved in this study.
Reporting on race, ethnicity, or other socially relevant groupings	Not applicable as no human participants are involved in this study.
Population characteristics	Not applicable as no human participants are involved in this study.
Recruitment	Not applicable as no human participants are involved in this study.
Ethics oversight	Not applicable as no human participants are involved in this study.

Note that full information on the approval of the study protocol must also be provided in the manuscript.

Field-specific reporting

Please select the one below that is the best fit for your research. If you are not sure, read the appropriate sections before making your selection.

[x] Life sciences   [ ] Behavioural & social sciences   [ ] Ecological, evolutionary & environmental sciences

For a reference copy of the document with all sections, see nature.com/documents/nr-reporting-summary-flat.pdf

Life sciences study design

All studies must disclose on these points even when the disclosure is negative.

Sample size

The RNAP MW was determined by the elution profiles obtained from size-exclusion chromatography and SDS-PAGE to confirm complex

	composition.<br>Protein concentration for both in vitro transcription assays and EM was measured using the Qubit 2.0 fluorimeter (invitrogen).<br>For EM, the RNAP diameter was experimentally determined on micrographs based on particles size.<br>Optimisation of nonspecific in vitro transcription reactions sampled a range of physiologically relevant conditions (pH, temperature, salt concentration, divalent cation type and concentration and DNA template types).
Data exclusions	No data were excluded.
Replication	For EM experiments, replications were not carried out other than sample optimization prior to data collection that allowed identification of the correct grids and sample concentration to use. Data from EM are rarely presented as replicates because replicates can affect data quality. Assay optimization and inhibitor screening were performed in triplicate and all attempts were successful.
Randomization	Cryo-EM: particles and their orientations are randomly distributed on each micrograph.<br>No randomization was applied to in vitro transcription assays to ensure the reproducibility of the experiments in the replicates.
Blinding	Blinding is not relevant because there are no animals or patients involved and all techniques used in this study require a precise identification and quantification of the proteins and reagents used, as well as strict control over buffer and experimental conditions applied to ensure the reproducibility of the results.

Reporting for specific materials, systems and methods

We require information from authors about some types of materials, experimental systems and methods used in many studies. Here, indicate whether each material, system or method listed is relevant to your study. If you are not sure if a list item applies to your research, read the appropriate section before selecting a response.

Materials & experimental systems

n/a	Involved in the study	
☒	☐	Antibodies
☐	☒	Eukaryotic cell lines
☒	☐	Palaeontology and archaeology
☒	☐	Animals and other organisms
☒	☐	Clinical data
☒	☐	Dual use research of concern
☒	☐	Plants

Methods

n/a	Involved in the study	
☒	☐	ChIP-seq
☒	☐	Flow cytometry
☒	☐	MRI-based neuroimaging

Eukaryotic cell lines

Policy information about cell lines and Sex and Gender in Research

Cell line source(s)	Insect cells used were Sf9 cells (ThermoFisher Scientific, Catalog No. 11496015) and High Five Cells (Invitrogen, Catalog No. B85502).
Authentication	Cells were from commercially available stocks, so were not authenticated.
Mycoplasma contamination	Cells were not tested for Mycoplasma contamination.
Commonly misidentified lines<br>(See ICLAC register)	No commonly misidentified cell lines were used in this study.
