# Supplementary figures and images for: Structure of the recombinant RNA polymerase from African Swine Fever Virus
Source: Nat Commun. 2024 Feb 21;15:1606. doi: 10.1038/s41467-024-45842-7 (PMC10881513; doi:10.1038/s41467-024-45842-7)

## Slide 1
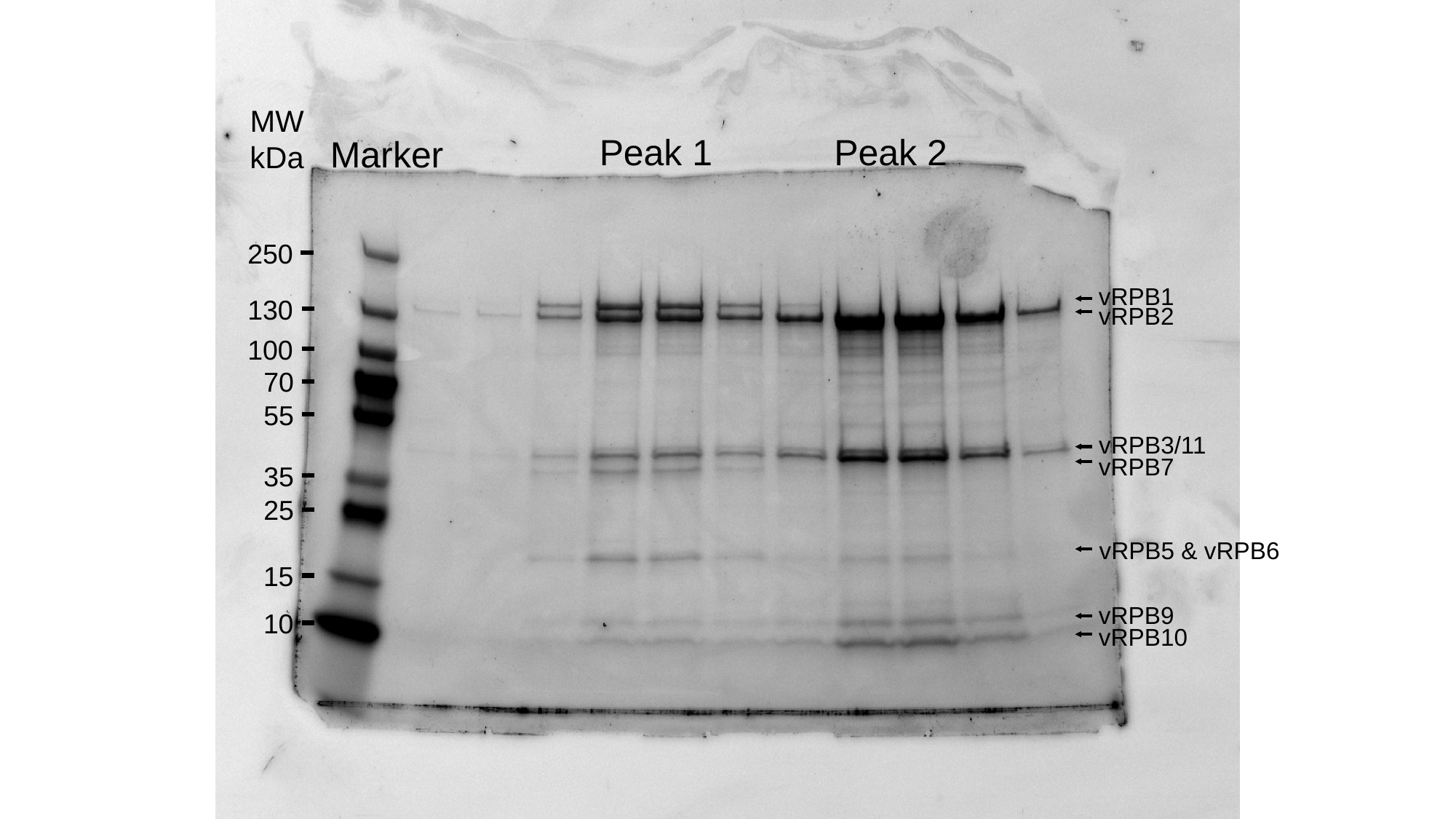

MW
kDa
Peak 1
Peak 2
Marker
250
vRPB1
130
vRPB2
100
70
55
vRPB3/11
vRPB7
35
25
vRPB5 & vRPB6
15
vRPB9
10
vRPB10

Supplement: Supplementary file 8 — Source Data [file 41467_2024_45842_MOESM8_ESM.zip › Source Data/Figure 1c source data.pptx]
